# Standardization of spray-dried powder of *Piper betle* hot water extract

**DOI:** 10.4103/0973-1296.80678

**Published:** 2011

**Authors:** Liyanage Dona Ashanthi Menuka Arawwawala, Horadugoda Gamage Sujatha Pushpakanthi Hewageegana, Lakshmi Sriyani Rajapaksha Arambewela, Hettiarachchige Sami Ariyawansa

**Affiliations:** *Industrial Technology Institute, Bauddhaloka Mawatha, Colombo 7, Sri Lanka*; 1 *Department of Nidana Chikitsa, Institute of Indigenous Medicine, University of Colombo, Rajagiriya, Sri Lanka*

**Keywords:** *Piper betle*, physicochemical parameters, phytochemicals

## Abstract

The leaves of *Piper betle* Linn. (Family: Piperaceae) possess several bioactivities and are used in the Traditional Medical systems of Sri Lanka. The present investigation was carried out to standardize the spray-dried powder of *P. betle* by (a) determination of physicochemical parameters, presence or absence of heavy metals, and microbial contamination; (b) screening for phytochemicals; and (c) development of High Pressure Liquid Chromatography (HPLC) fingerprint and densitogram. The percentages of moisture content, total ash, acid insoluble ash, water-soluble ash, and ethanol extractable matter of spray-dried powder of *P. betle* were 2.2-2.5, 6.8-7.0, 0.003-0.005, 4.1-4.3, and 15.8-16.2, respectively. The concentrations of all the tested heavy metals were below the WHO acceptable limits and bacterial species, such as *Escherichia coli, Salmonella* spp, *Staphylococcus aureus*, and *Pseudomonas aeroginosa* were not present in the *P. betle* spray-dried powder. Phenolic compounds, tannins, flavonoids steroids, and alkaloids were found to be present in the spray-dried powder of *P. betle* and HPLC fingerprint and densitogram clearly demonstrated the proportional differences of these chemical constituents. In conclusion, the results obtained from this study can be used to standardize the spray-dried powder of *P. betle*.

## INTRODUCTION

Plant-based drugs have been in use against various diseases since time immemorial. The primitive man used herbs as therapeutic agents, which they were able to procure easily.[1] *Piper betle* Linn. (Family: Piperaceae) is a perennial dioecious, semi-woody climber and cultivated in Sri Lanka, India, Malaysia, Indonesia, Philippine Islands, and East Africa. Stems are strongly swollen at the nodes, papillose when young, soon entirely glabrous. Leaves alternate, simple, and yellowish green to bright green in color. Leaves of fertile branches with a petiole 1-2 cm long, 1.2-1.8 mm thick when dry and glabrous at maturity.[2,3] More than 12 *P. betle* cultivars are reported in Sri Lanka[4] and except cultivar called Malabulath, which is not used for chewing, other cultivars constitute the “commercial betel” of Sri Lanka. According to Kumaratunga,[5] chemical constituents and their relative proportions in essential oil of “commercial betel” of Sri Lanka are different from those of other countries. In Sri Lanka, research work has been carried out to evaluate some selected bioactivities, such as antimotility effects on washed human spermatozoa,[6] antiaphrodisiac activity,[7] antifertility effects of male rats,[8] antimicrobial,[3] antidiabetic,[9] antinociceptive[10] and antioxidant[11] activities using hot water extract of *P. betle* leaves.

In modern herbal drug industry, there is a trend to manufacture tablets and capsules from spray-dried powders of bioactive plant extracts. Examples include Amla spray-dried powder, Neem spray-dried powder, Arjuna spray-dried powder, Turmeric spray-dried powder, Noni spray-dried powder, *Aleo vera* spray-dried powder and so on. Therefore, an attempt was made to prepare a spray-dried powder from *P. betle* hot water extract and standardize the spray-dried powder by (a) determining the physicochemical parameters, (b) screening the phytochemical constituents, and (c) developing the High Pressure Liquid Chromatography (HPLC) fingerprints and densitograms.

## MATERIALS AND METHODS

### Plant material

*P. betle* leaves were purchased from the main vegetable markets in the Western province of Sri Lanka. The leaves were identified and authenticated by the Curator of National Herbarium, Royal Botanical Gardens, Peradeniya, Sri Lanka. A voucher specimen (PS 01) was deposited in the Industrial Technology Institute, Colombo 7, Sri Lanka.

### Preparation of spray-dried powder from hot water extract of *Piper betle*

*P. betle* leaves were air dried for 3-5 days in the shade, cut into small pieces and 20 kg were boiled with 100 L of distilled water for 4 h. The extract was filtered and the filtrate was concentrated under vacuum until the total solid content exceeded more than 25%. The concentrated extract was further subjected to a spray dryer (Kestner Patent Spray Dryer, made in UK; inlet: 175°C and outlet: 100°C) to obtain the powder form the extract.

### Determination of physicochemical parameters of *Piper betle* spray-dried powder

Physicochemical parameters such as moisture content, total ash content, acid insoluble and water soluble ash contents and ethanol extractable matter were determined for *P. betle* spray-dried powder according to the methods described in guidelines of WHO.[12]

### Determination of moisture content

The powdered material (1 g) was placed in a moisture dish and dried to a constant weight in an oven at 100 °C-105 °C. The loss of weight in mg/g of air-dried material was calculated.

### Determination of total ash content

The powdered material (2 g) was accurately weighed and placed in a crucible. The material was ignited to a constant weight by gradually increasing the heat to 500 °C-600 °C until it was white. The residual ash was allowed to cool in a desicator. The content of total ash in mg/g of air dried material was calculated.

### Determination of acid insoluble ash content

Hydrochloride acid (2 N; 25 mL) was added to the crucible containing the total ash, covered with a watch glass and boiled gently for 5 min. The watch glass was rinsed with 5 mL of hot water and the rinsed contents were added to the crucible. The acid insoluble matter was collected on an ashless filter paper and washed with hot water until the filtrate was neutral. The filter paper containing acid insoluble matter was transferred to the original crucible, dried and ignited to a constant weight. The residue was allowed to cool in a desicator and weighed. The content of the acid insoluble ash in mg/g of air dried material was calculated.

### Determination of water soluble ash content

Water (25 mL) was added to the crucible containing the total ash, covered with a watch glass and boiled gently for 5 min. The watch glass was rinsed with 5 mL of hot water and added to the crucible. The water insoluble matter was collected on an ashless filter paper and washed with hot water. The filter paper containing the water insoluble matter was transferred to the original crucible, dried on a hot plate and ignited to a constant weight. The water soluble ash content was calculated.

### Determination of ethanol extractable matter

Accurately weighed powdered material (4 g) was placed in a glass stoppered conical flask. Ethanol (95%; 100 mL) was added to the flask and it was weighed to obtain the total weight including the flask. Then, the flask was shaken well and kept for 1 h. A reflux condenser was attached to the flask and boiled gently for 1 h, and then it was cooled and weighed. The weight was readjusted to the original total weight by adding required amount of 95% ethanol. The contents were filtered rapidly. After that, 25 mL of the filtrate was evaporated to dryness on a water bath. Then the dish was dried at 105 °C for 6 h, cooled in a desiccator, and weighed. The content of extractable matter in mg/g air dried material was calculated.

### Determination of heavy metals in the spray-dried powder

Presence or absence of heavy metals (Hg, Pb, Cd, and As) were determined according to SLS[13] and AOAC[14] standards.

### Determination of microbial contamination in the spray-dried powder

Presence or absence of *Escherichia coli, Salmonella* spp, *Staphylococcus aureus* and *Pseudomonas aeroginosa* were determined according to the standards of SLS.[15–17]

### Preliminary phytochemical screening of *Piper betle* spray-dried powder

The qualitative chemical tests were performed for the *P. betle* spray-dried powder according to the methods described by Farnsworth[18] with some modifications.

#### Determination of presence/absence of phenolic compounds

Two to three drops of 1% FeCl_3_ solution was added into 2 mL portions (1%) of the spray-dried powder. Phenolic compounds produce a deep violet color with ferric ions.

#### Determination of presence/absence of tannins

The spray-dried powder was diluted with water and added to diluted FeCl_3_ solution. Tannins give a blackish blue or green blackish color in the presence of FeCl_3_.

#### Determination of presence/absence of flavonoids

The spray-dried powder was dissolved in methanol (50%, 1-2 mL) by heating. Then metal magnesium and 5-6 drops of conc. HCl were added. The solution turns red when flavonoids are present.

#### Determination of presence/absence of steroid glycosides

The spray dried powder was dissolved in equal volumes of acetic anhydride and CHCl_3_. The mixture was transferred to a dry test tube and conc. H_2_SO_4_ acid was added at the bottom of the tube. Formation of a reddish brown or violet-brown ring at the interface of the two liquids indicates the presence of steroids.

#### Determination of presence/absence of alkaloids

The alkaloids were extracted by refluxing the sample with sufficient amount of water for about 2 h. The extract was concentrated, adjusted to a high pH (pH = 9.2) with NH_4_OH and was extracted with CHCl_3_ (three times). Then the content was concentrated and 2 drops were spotted separately on a Thin Layer Chromatography plate. After the spots were dried, Dragendorff’s reagent was sprayed onto them. Alkaloids give an orange color with Dragendorff’s reagent.

### Development of HPLC fingerprint

#### Sample preparation

*P. betle* spray-dried powder (10 mg) was dissolved in 10 mL of distilled water and purified using Sep-Pak C_18_ cartridge.

Injection volume : 20 µL

Apparatus : Waters 501 HPLC pump SPD 10AV UV-vis detector (Shimadzu, Japan)

Column : Hypersil^®^ ODS (C_18_)

Solvent system : Acetonitrile:Water (1:1)

Flow rate : 1 mL/min

Detection : 254 nm

### Development of densitogram

#### Sample preparation

*P. betle* spray-dried powder (10 mg) was dissolved in 10 mL of distilled water and 5 µL was spotted on Silica Gel_254_

Absorbent : Silica Gel_254_

Solvent system : Butanol:Methanol:Water (1:1:0.2)

Scanning : Densitometer (CS-9301PC, Shimadzu, Japan) at 254 nm.

## RESULTS

### Physicochemical parameters

Results are listed in Table 1.

**Table 1:** Physicochemical parameters of spraydried powder of *Piper betle* hot water extract

Moisture content	2.2%-2.5% (w/w)
Total ash	6.8%-7.0% (w/w)
Acid insoluble ash	0.003%-0.005% (w/w)
Water soluble ash	4.1%-4.3% (w/w)
Ethanol extractable matter	15.8%-16.2% (w/w)
Heavy metals^a^	
Mercury (Hg)	Not detected
Lead (Pb)	Not detected
Cadmium (Cd)	Not detected
Arsenic (As)	Not detected
Microbial contamination	
Test for *Escherichia coli*/g	Absent
Test for *Salmonella* spp/g	Absent
Test for *Staphylococcus aureus*/g	Absent
Test for *Pseudomonas aeroginosa*/g	Absent

a Limit of detection (mg/kg); Pb: 0.50; Cd: 0.10; As: 0.05; Hg: 0.05

### Phytochemical screening studies

Phytochemical screening studies revealed the presence of phenolic compounds, tannins, flavonoids, steroids and alkaloids in the spray-dried powder of *P. betle* hot water extract.

### HPLC fingerprint and densitogram

**Figure 1:**
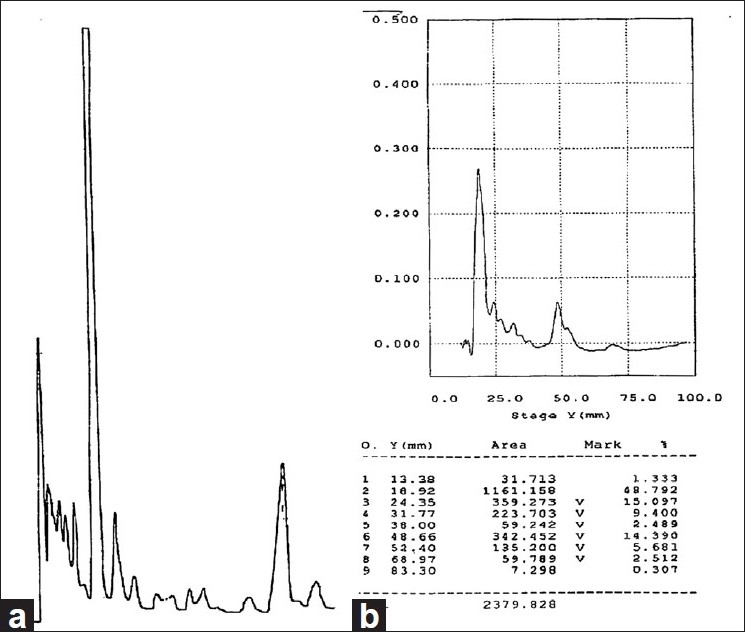
(a) HPLC fingerprint; and (b) densitogram of spray-dried powder of *Piper betle* hot water extract

As shown in Figure 1, spray-dried powder of *P. betle* hot water extract was standardized by using HPLC fingerprint and densitogram.

## DISCUSSION

The residue remaining after incineration of plant material is the ash content or ash value, which simply represents inorganic salts, naturally occurring in crude drug or adhering to it or deliberately added to it, as a form of adulteration. The ash value was determined by 3 different methods, which measured total ash, acid insoluble ash, and water soluble ash. The total ash is used to measure the total amount of material remaining after ignition. This includes both “physiologic ash,” which is derived from the plant tissue itself, and “nonphysiologic ash,” which is the residue of the extraneous matter adhering to the plant surface. Acid insoluble ash measures the amount of silica or acid insoluble matter present. Water soluble ash is the water soluble portion of the total ash. These ash values are important quantitative standards.[19]

Extractive values are representative of the presence of the polar or nonpolar compounds in a plant material. The water soluble extractive of ginger is expected to be in the range of the 10% with respect to air dried material, lowering of this extractive value indicates the addition of exhausted material with the original drug. Moisture is an inevitable component of crude drugs, which must be eliminated as far as practicable. Insufficient drying favors spoilage by molds and bacteria and enzymatic destruction of active principles.[19,20]

The presence or absence of 4 heavy metals, namely, As, Hg, Cd, and Pb, was analyzed in the *P. betle* spray-dried powder and results are shown in Table 1. The concentrations of all the tested heavy metals were below the WHO acceptable limits. As shown in Table 1, bacterial species, such as *Escherichia coli, Salmonella* spp, *Staphylococcus aureus*, and *Pseudomonas aeroginosa* were not found in the *P. betle* spray-dried powder. Therefore, this is a good indicator of the hygiene of the preparation.

Standardization of herbal products/drugs is more challenging than synthetic drugs. Herbal extracts contain number of constituents of complex chemical nature and are inconsistent in composition.[21] In most of the cases the biological activity is not exclusively dependent on the so-called active constituents, but is due to synergistic effect of all the chemical constituents of the plant. Even though biologically inert, many constituents affect the pharmacokinetics and stability of the active constituents.[22] Phytochemical screening revealed the presence of phenolic compounds, tannins, flavonoids, steroids, and alkaloids in the *P. betle* spray-dried powder and standardized by using a HPLC fingerprint and a densitogram.

In conclusion, the results obtained from physicochemical parameters, phytochemical screening studies and development of HPLC fingerprint and densitogram can be used to standardize *P. betle* spray-dried powder.
